# Investigating Viral Inoculation and Recovery from Medical Masks

**DOI:** 10.1155/2022/3173883

**Published:** 2022-02-21

**Authors:** Mark C. Wilkinson, Jennifer Carney

**Affiliations:** ^1^Sanitas Healthcare, Bateman House, 82-88 Hills Road, Cambridge, Cambridgeshire CB2 1LQ, UK; ^2^Virologica, ITAC Bio, Sci-Tech Daresbury, Keckwick Lane, Warrington, Cheshire WA4 4AD, UK

## Abstract

The SARS-CoV-2 pandemic from 2019 onwards has significantly increased the usage of surgical style medical masks, both in healthcare and public settings. It is important to study the contamination of and viral transfer from such masks. However, accepted standard test methods such as ISO 18184 have prescribed inoculation methods which may not be fully representative of the type of viral insult experienced in the clinic or community. In addition to studying a conventional mask, the performance of a mask featuring an antimicrobial photosensitiser was also studied.

## 1. Introduction

Recently, the SARS-CoV-2 pandemic has significantly increased the usage of surgical style masks, both in healthcare and public settings. Not only has their use proliferated but also the typical duration of wear has increased significantly. Widespread use by nonhealthcare professionals has also eroded best practice regarding touching of contaminated masks and potential reuse of masks. This scenario raises several questions that could be addressed via laboratory experimentation. However, accepted methods for the study of viral contamination of fabrics—such as ISO 18184—describe standard microbiological techniques which may not best represent the presentation of viruses or the use of such masks in the clinic or community.

Duration of mask wearing has increased, so repeat inoculation over extended time periods was studied. Standard methods such as ISO 18184 require direct inoculation with pipetted drops of viral suspension. However, in the real world, virus is spread as fine droplet suspensions or aerosols. To better represent this, we challenged a mask with an aerosolised inoculum.

Medical (or surgical) masks typified by the type I/II/IIR classification in Europe are constructed from multiple layers of nonwoven fabric—normally between 3 and 5 layers—bonded together. Previous work has found a build-up of respiratory viruses on the outer layer of surgical type masks [1].

Indeed, the consensus standard for such devices EN 14683 : 2019 regards the mask outers as “highly contaminated” after use. SARS-CoV-2, in particular, has been shown to survive well on surgical masks, being still detectable 7 days after exposure [2]. Thus, the transfer of virus from contaminated masks was studied.

In addition, the performance of an antimicrobial photosensitiser which has found success in other medical devices was studied here in an antiviral mask.

## 2. Materials and Methods

### 2.1. Masks

Standard surgical style type IIR masks (code PM-PG4-1001) were supplied by Primed Medical Products, Edmonton, Canada. The antiviral mask was the Protect Antiviral Type IIR Mask supplied by Sanitas Healthcare, UK. In experiments designed to determine antiviral efficacy of the Protect Antiviral Type IIR mask, the same mask was used as control with the antiviral outer layer removed, with the exception of the aerosol study where a standard mask was used as a control.

### 2.2. Viral Aerosol

An Intranasal Mucosal Atomization Device (IMAD) was used to deliver 300 *µ*l of a viral inoculum in aerosol form. The device was the MAD Nasal™ supplied by Teleflex, fitted to a 1 ml syringe. The MAD Nasal™ is stated by the manufacturer to deliver a fine mist of particles 30–100 microns in size.

### 2.3. Cells and Virus

MRC-5 pd19 cells (ECACC 05072101) were cultured in Eagle's minimum essential medium (EMEM) supplemented with 10% (vol/vol) foetal bovine serum (FBS) at 37°C in 5% CO_2_. Virus used was human coronavirus 229E (HCoV-229E) obtained from the National Collection of Pathogenic Viruses (NCPV 0310051v). Working virus stocks were generated by infecting MRC-5 cells at a multiplicity of infection (MOI) of 0.01 in the absence of FBS and incubating at 33°C under a 5% CO_2_ atmosphere until cytopathic effect (CPE) was observed in more than 80% of cells (4-5 days after infection). Cell culture supernatants were clarified by centrifugation at 1000x g for 10 minutes and aliquoted before storage at −80°C. When concentrated virus inoculum was required, clarified culture supernatant was concentrated using VivaSpin^TM^ 20 (100 kDa cut off) centrifugal concentrators.

### 2.4. Antiviral Efficacy

Antiviral efficacy was tested following the standard test method ISO 18184 with noted modifications. ISO 18184 stipulates 200 *µ*l inoculum to be applied to the test sample at time zero. During the aerosol study, a total of 300 *µ*l inoculum was applied to the mask over a period of 3 hours (applied hourly after the initial inoculation, exposed to approx. 1000 lux). Following the final inoculation, the mask was held for 1 hour before being transferred to 20 ml of neutraliser (recipe as per ISO 18184; 17 g/l casein peptone, 3 g/l soybean peptone, 5 g/l sodium chloride, 2.5 g/l dipotassium hydrogen phosphate, 2.5 g/l D-glucose, 1 g/l lecithin, and 7 g/l Tween 80). Any remaining viable virus was recovered by vortexing for 5 × 5 seconds and quantified. In the repeated inoculation study, 300 *µ*l of inoculum was applied over a period of 8 hours (75 *µ*l applied at time zero and then 75 *µ*l applied every 2 hours, exposed to approx. 1000 lux). Following the final inoculation, the mask was held for 2 hours before being transferred to 20 ml of neutraliser. Any remaining viable virus was recovered by vortexing for 5 × 5 seconds and quantified.

### 2.5. Transfer Study

To demonstrate the potential to reduce transfer of viable virus from contaminated mask surfaces, approx. 4 cm × 4 cm samples of Protect Antiviral Type IIR mask and control mask were inoculated dropwise, distributing multiple (*ca*. 18) drops evenly across the surface of the sample, to a final volume of 200 *µ*l of virus. After 1 hour incubation at 25°C (exposed to approx. 1000 lux), 5 pieces of nitrile glove (approx. 2 cm × 2 cm) were pressed on to the mask sample, each in a different area to ensure that the entire mask surface had been touched at least once and transferred to 10 ml EMEM. Any viable virus that had been transferred to the glove was recovered by vortexing for 5 × 5 seconds and quantified. The gloves used were Pulse® Nitrile, manufactured by Innovative Healthcare Corporation. To ensure a consistent surface, the gloves were washed with water before use and allowed to dry.

### 2.6. Virus Quantification

Viral titres and viral reductions were determined by TCID_50_ assays. MRC-5 cells were seeded into 96 well plates (1 × 10^4^ cells/well) 1-2 days before the assay. Ten-fold serial dilutions of sample were prepared in serum-free EMEM, and each dilution was inoculated into 8 wells of a 96-well plate (100 *µ*l/well). Plates were incubated for 1-2 hours at 33°C in 5% CO_2_. Following incubation for all assays except antiviral efficacy testing, 100 *µ*l of serum free media was added to the wells. For antiviral efficacy testing, following incubation, inoculum was removed and wells were washed with 100 *µ*l of serum-free EMEM before being replaced with 200 *µ*l serum-free EMEM. Plates were incubated at 33°C in 5% CO_2_ for 7 days. TCID_50_ titres were determined by the Behrens and Karber method as detailed in ISO 18184 [3].

## 3. Results

### 3.1. Standard Inoculation according to ISO18184

Masks were inoculated with 200 *µ*l of a 4.22 × 10^6^ TCID_50_/ml inoculum of HCoV-229E, thus 8.43 × 10^5^ TCID_50_/mask. Immediate recovery of virus from the mask (*T* = 0) yielded 7.38 × 10^5^ TCID_50_/mask, representing greater than 87% recovery. Following 1 h of incubation, a >3 log reduction (99.91%) was observed from the antiviral mask compared to the control at *T* = 0 (as per ISO 18184). The comparison to the control at *T* = 1 h (99.73% reduction) is included for information. Neutraliser induced CPE present in all neat wells prevented viral quantification below 6.32 × 10^2^ TCID_50_/mask.

### 3.2. 8-Hour Repeated Inoculation

Masks were inoculated with 300 *µ*l of a 4.22 × 10^7^ TCID_50_/ml inoculum of HCoV-229E, thus 1.27 × 10^7^ TCID_50_/mask. *A* >4 log reduction (99.99%) was observed from the antiviral mask at 8 hours compared to the control at *T* = 0 (as per ISO 18184).

### 3.3. MAD Aerosol Inoculation

Masks were inoculated with 300 *µ*l of a 1.00 × 10^7^ TCID_50_/ml inoculum of HCoV-229E, thus 3.00 × 10^6^ TCID_50_/mask. *A* >3 log reduction (99.97%) was observed from the AV mask at 4 hours compared to the control at *T* = 0 (as per ISO 18184).

### 3.4. Transfer Study

Masks were inoculated with 200 *µ*l of a 3.16 × 10^7^ TCID_50_/ml inoculum of HCoV-229E, thus 6.32 × 10^6^ TCID_50_/mask. The results are reported “per glove” as the virus is recovered from a glove, after transfer from the mask.

**Figure 1:**
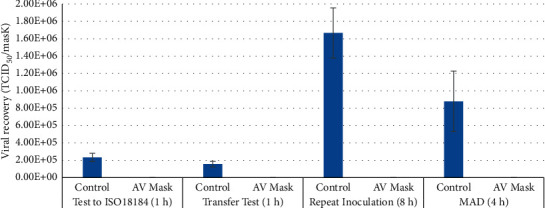
Viral recovery from standard (“control”) type IIR mask vs. photosensitiser-treated antiviral (AV) type IIR mask at stated times. The experiments compared are (a) standard ISO 18184 protocol, (b) viral transfer test via medical examination glove, (c) repeat inoculation with virus every 2 h for 8 h duration, and (d) aerosol inoculation via MAD device, over 4 h.

A visual representation of the above results is presented in Figure 1.

## 4. Discussion

### 4.1. Standard Inoculation according to ISO 18184

Our initial experiments used standard techniques as described in ISO 18184. We used this precedented method to validate simple inoculation and recovery of human coronavirus 229E from medical facemasks. A recovery greater than 87% of the initial inoculum was demonstrated immediately following inoculation of the control mask confirming suitability of the test method (Table 1).

To potentially mitigate infectious viral contamination, the utility of an antimicrobial photosensitiser was studied. A cationic zinc phthalocyanine which has previously shown good activity in antimicrobial gloves was chosen [4]. This molecule is from a class of photosensitisers known to transfer visible light energy to atmospheric oxygen, yielding the short-lived activated singlet oxygen. Singlet oxygen is known to be a potent antimicrobial agent, with excellent activity against enveloped viruses such as SARS-CoV-2 and HCoV-229E [5, 6]. In addition, the cationic nature of this photosensitiser gave it good affinity for the nonwoven cellulose layer of the mask under study. This excellent affinity was demonstrated when the mask was extracted with water for 72 hours at 37°C as per ISO 10993-12, and no photosensitiser was detected by UV/Vis spectroscopy of the extract (to the limit of detection).

This bound, nonleaching presentation of the photosensitiser reduces risk to the wearer. In addition, due to the short half life and diffusion range of singlet oxygen, any biocidal activity of the mask is expressed at the surface only and not in the wider environment. During incubation, the test and control masks were illuminated with ca. 1000 lux of white LED light. This light intensity was taken from standard EN 12464-1 as the recommended intensity for medical examination.

When this antiviral mask (the protect antiviral type IIR mask) was investigated using a typical ISO 18184 protocol, a >99.9% reduction in HCoV-229E was recorded at 1 hour (Table 1). It is possible that a significantly greater log reduction had been achieved, but due to cytotoxicity caused by the neutraliser, CPE was observed in the neat wells for all samples. It was not possible to distinguish any viral CPE from neutraliser induced CPE; therefore, these wells had to be recorded as positive, limiting the calculated log reduction values. No CPE and thus no virus were detectable in any wells beyond the neat sample for all antiviral mask samples.

### 4.2. 8 Hour Repeated Inoculation

As an established laboratory virology test method for textiles, ISO 18184 offered a credible starting point for our studies. However, this method requires only a single inoculation. With pandemic conditions, bringing mask wearers more commonly into contact with viral challenge, it was desirable to study the impact of repeat inoculations. A control mask was inoculated with 75 *µ*l of HCoV-229E inoculum every 2 hours for 8 hours. The suitability of the recovery method and the ability of the virus to survive under the test conditions were demonstrated (Table 2).

When the repeat inoculum study was performed on the mask featuring an outer cellulose layer treated with the antiviral photosensitiser, a significant 99.99% reduction in virus was recorded at the end of the 8 hour study. This demonstrated the ability of the mask to continue to respond to viral insult throughout its duration of wear by maintaining antiviral activity. A comparison between these data and our other results is represented in Figure 1.

### 4.3. MAD Aerosol Inoculation

We felt there were limitations to the inoculation method according to ISO 18184, which uses large aliquots pipetted on to test articles. Person to person viral spread is more typically in the form of fine droplet suspensions or aerosols. We identified the potential utility of an intranasal mucosal atomization device to deliver aerosolised inoculum. The Teleflex MAD Nasal™ was used, which is stated by the manufacturer to deliver a fine mist of droplets in the range of 30 to 100 microns. This device has been used to more realistically mimic viral challenge in animal models, including SARS-CoV-2 [7, 8].

The protocol was validated by inoculating a standard mask hourly for 4 hours, using the IMAD. The total volume of the starting inoculum (300 *µ*l of 1.00 × 10^7^ TCID_50_/ml HCoV-229E) was delivered to the mask in this period. The viral recovery from the mask at the end of the experiment was 2.34 × 10^6^ TCID_50_/mask (Table 3). Thus, the validity of the inoculation pathway and recovery was demonstrated.

Next, we studied the performance of the antiviral mask using the aerosol inoculation method. After application of an aerosol inoculum to masks featuring an outer cellulose layer treated with the antiviral photosensitiser, a 99.97% reduction of HCoV-229E was observed (Table 3). Thus, the delivery of a more representative inoculation still showed a significant antiviral effect.

### 4.4. Transfer Study

In addition to considering inoculation—how virus is placed on a mask—we also evaluated cross contamination—how virus may subsequently spread from a contaminated mask surface.

Medical masks should not be touched during wear, and prescribed removal procedures should be followed. In reality, best practice is not always rigorously adhered to. This problem has become compounded during the coronavirus pandemic since duration of wear has significantly increased for both professional and public use. Touching the outside of the mask to adjust fit and tightening a nose band or to eat and drink are all plausible scenarios where this can occur.

With the risk for touching of masks during use established, we considered what hazard might result. The consensus standard for such medical masks EN 14683 : 2019 details that postuse masks should be considered “highly contaminated.” As previously stated, it has been shown that respiratory viruses concentrate on masks during use [1]. It has also been shown that, during routine removal, viral contamination can be efficiently transferred from PPE (including masks) to the wearer's skin or clothing [9].

To mimic the use of masks by healthcare professionals, we studied viral transfer from masks to medical examination gloves. Cross contamination of pathogens from surfaces via gloves has been studied previously, and we adopted these principals in our work [10].

The viability of the protocol to efficiently transfer virus was demonstrated with 1.56 × 10^5^ TCID_50_/glove being transferred from an inoculated control mask, after 1 h of incubation. In contrast, when the same transfer study was performed with the mask featuring the photosensitiser, no viable virus was detected in the assay (Table 4). Thus, the potential positive impact of the antimicrobial photosensitiser to prevent cross contamination from masks was demonstrated.

## 5. Conclusion

In conclusion, the inoculation of surgical style medical masks has been investigated, deploying techniques more representative of real mask use than established lab protocols. Successful recovery of virus has been demonstrated, validating the protocols. In addition, the utility of an antimicrobial photosensitiser to deactivate virus on the mask outer has been established. This could have a potential positive impact in reducing viral cross contamination from such masks.

## Figures and Tables

**Table 1:** Viral recovery from standard (“control”) type IIR mask vs. photosensitizer-treated antiviral (AV) type IIR mask at stated times, according to international standard ISO 18184.

Mask	Replicate	TCID_50_/mask	Average TCID_50_/mask	% reduction of antiviral mask	SD	Significance
Control, *T* = 0	1	6.32 × 10^5^	7.38 × 10^5^	99.91	±1.49 × 10^5^	*p* < 0.01
	2	8.43 × 10^5^				
Control, *T* = 1 h	1	2.67 × 10^5^	2.34 × 10^5^	99.73	±4.74 × 10^4^	*p* < 0.05
	2	2.00 × 10^5^				
Antiviral, *T* = 1 h	1	≤6.32 × 10^2^	≤6.32 × 10^2^			
	2	≤6.32 × 10^2^				

**Table 2:** Viral recovery from standard (“control”) type IIR Mask vs. photosensitizer-treated antiviral (AV) type IIR mask, with 4 repeated inoculations at 2 hour intervals and an 8 hour total experiment duration.

Mask	Replicate	TCID_50_/mask	Average TCID_50_/mask	% reduction of antiviral mask	SD	Significance
Control, *T* = 0	1	8.43 × 10^6^	1.03 × 10^7^	99.99	±1.60 × 10^6^	*p* < 0.001
	2	1.12 × 10^7^				
	3	1.12 × 10^7^				
Control, *T* = 8 h	1	1.50 × 10^6^	1.67 × 10^6^	99.96	±2.89 × 10^5^	*p* < 0.001
	2	2.00 × 10^6^				
	3	1.50 × 10^6^				
Antiviral, *T* = 8 h	1	≤6.32 × 10^2^	≤6.32 × 10^2^			
	2	≤6.32 × 10^2^				
	3	≤6.32 × 10^2^				

**Table 3:** Viral recovery from standard (“control”) type IIR mask vs. photosensitizer-treated antiviral (AV) type IIR mask at stated times, after inoculation with a viral suspension provided by the Teleflex MAD Nasal™ device.

Mask	Replicate	TCID_50_/mask	Average TCID_50_/mask	% reduction of antiviral mask	SD	Significance
Control, *T* = 0	1	2.67 × 10^6^	2.34 × 10^6^	99.97	±4.74 × 10^5^	*p* < 0.01
	2	2.00 × 10^6^				
Control, *T* = 4 h	1	6.32 × 10^5^	8.76 × 10^5^	99.93	±3.45 × 10^5^	*p* < 0.05
	2	1.12 × 10^6^				
Antiviral, *T* = 4 h	1	≤6.32 × 10^2^	≤6.32 × 10^2^		0	
	2	≤6.32 × 10^2^				

**Table 4:** Viral recovery from medical examination gloves, which had been used to transfer virus from the surface of a standard (“control”) type IIR mask or a photosensitizer-treated antiviral (AV) type IIR mask.

Mask	Replicate	TCID_50_/glove	Average TCID_50_/glove	% reduction of antiviral mask	SD	Significance
Control	1	1.33 × 10^5^	1.56 × 10^5^	99.99	±3.18 × 10^4^	*p* < 0.05
	2	1.78 × 10^5^				
Antiviral	1	0	0			
	2	0				

## Data Availability

The data used to support the findings of this study are available from the lead author (MW) upon request.
